# Challenges and solutions for implementing telemedicine in Iran from health policymakers’ perspective

**DOI:** 10.1186/s12913-023-10488-6

**Published:** 2024-01-10

**Authors:** Seyed Mojtaba Hosseini, Shiva Abdolahnejad Boushehri, Khalil Alimohammadzadeh

**Affiliations:** 1 Department of Health Services Management, North Tehran Branch, Islamic Azad University, Tehran, Iran; 2 Health Economics Policy Research Center, Tehran Medical Branch, Islamic Azad University, Tehran, Iran

**Keywords:** Challenges, Solutions, Implementing, Telemedicine

## Abstract

**Background:**

Despite significant progress in health technology and growing interest among countries in incorporating telemedicine into healthcare delivery, its usage remains limited in Iran. The aim of this study is to investigate the challenges related to telemedicine in Iran and pinpoint potential solutions from the viewpoint of health policymakers, marking the first such endeavor.

**Methods:**

This qualitative study was conducted in Iran in 2022. Data were gathered from 19 health policymakers who were selected using purposeful and snowball sampling techniques via in-depth and semi-structured interviews. The research findings were analyzed using the content analysis technique, with coding performed using MAXQDA software. The content analysis approach developed by Erlingsson was utilized to analyze the data.

**Results:**

The study revealed eight main challenges that inhibit the widespread use of telemedicine in Iran. These challenges include policy weaknesses, uncertainty around operating mechanisms, inadequate communication and telecommunication infrastructure, insufficient cultural infrastructure, lack of electronic requirements, redundant bureaucracies, legal gaps, and economic factors. Furthermore, four key solutions to these challenges were identified. These include a national commitment to the development of telemedicine, the establishment of a telemedicine roadmap, the enhancement of e-health requirements and infrastructure, and the preparation of the community to accept telemedicine as a viable option for healthcare delivery.

**Conclusion:**

The implementation of telemedicine in Iran faces significant challenges, some of which are related to the national healthcare system, while others stem from various policy-related institutions and organizations. Addressing these challenges will require extensive inter-organizational cooperation and strong leadership at the governance level. However, it should be noted that fully resolving these issues is a time-consuming process.

**Supplementary Information:**

The online version contains supplementary material available at 10.1186/s12913-023-10488-6.

## Introduction

Most countries are grappling with a shortage of healthcare providers and an aging population. The challenges of living in remote areas, coupled with high transportation costs, often restrict access to medical services compared to those residing in urban or developed regions. These limitations intensify the situation, especially during emergencies that demand immediate response [1].

The healthcare sector employs telemedicine to overcome such barriers and increase access to health services. Significant advancements in information and communication technology, especially web-based technologies, provide new capabilities in delivering better health services to the population. Telemedicine is gradually emerging as a practical policy option in developing country governments [2]. The rationale behind it is to reduce obstacles by augmenting facilities and decreasing limitations [3].

Telemedicine is indeed a transformative technology in the healthcare sector. It covers a wide range of services, from healthcare education and clinical care delivery to administrative services and even remote home care. As such, healthcare providers and researchers view the development of telemedicine as a crucial component in managing health services and information systems [4].

According to studies, the United States, United Kingdom, and Canada lead in providing health services using telemedicine [5]. For instance, 60% of U.S. health care providers use telemedicine technology, even through remote counseling. This number is 57% in the United Kingdom and 45% in Canada [6]. Despite its potential, examining the use of telemedicine services reveals that policymakers recommended its use and providers and recipients of healthcare services still underutilize it [7]. Many barriers to implementing telemedicine remain persistent over time, with little progress being made despite continued research in this area [8].

In Iran, due to geographical conditions like mountainous regions, impassable deserts, inadequate transportation networks, population dispersion, limited medical facilities in deprived areas, the concentration of health facilities in metropolitan areas, and high transportation costs, telemedicine presents an efficient option. It can expedite disease diagnosis, facilitate appropriate medical techniques, reduce wasted time, lower diagnostic fees, and connect specialized hospitals in different cities in Iran [9].

The recent COVID-19 pandemic highlights the importance of agile responses and robust public health-related capabilities within health information systems [10]. Therefore, assuming telemedicine will not be a short-term issue but will experience further improvement and emerging approaches, it is crucial to consider it an efficient policy option. Telemedicine can help manage recent epidemics and create a bright and robust future for new generations [11].

Given the benefits and opportunities that telemedicine implementation presents for a developing country like Iran, it is clear that there are still obstacles to its successful deployment. This study examines and identifies telemedicine implementation challenges from a health policymaker’s perspective in Iran. The study seeks to provide operational solutions, remove barriers, benefit community members, and improve health indicators at the national level. The utilization of the perspective of health policymakers in scrutinizing challenges and proposing solutions is a novelty of this research.

## Materials and methods

This research is a qualitative descriptive cross-sectional study carried out in Iran in 2022. The study employed purposive sampling initially, which was then supplemented by the snowball method during the research process. The participants were selected based on their expertise in telemedicine policy within health organizations. Data saturation was achieved after conducting 19 interviews. In total, 19 policymakers participated, representing various roles in health service provision, including treatment, hygiene, development, research, and technology. Participants were sourced from the health ministry and its affiliated universities of medical sciences, the Health Insurance Organization, and the National Medical Informatics Association.

Research data were collected using deep and semi-structured face-to-face interviews. Initially, depth interviews were conducted with the purposive sampling approach. The main question of the in-depth interview was: What are the challenges associated with the implementation of telemedicine, and what solutions can be proposed to facilitate its deployment in Iran?. After conducting a content analysis of the interviews, the process concluded with semi-structured interviews. All interviews were conducted between April and July 2022, with interview durations ranging from 20 to 85 min. At the beginning of each interview, participants were informed of the study’s purpose, and informed consent was obtained. The recorded interviews were transcribed and analyzed using content analysis approach and MAXQDA 2018. The content analysis method was based on suggestions by Erlingsson and colleagues [12]. In the Erlingsson method, the process of data analysis begins with reading the entire dataset continuously to gain a deep understanding of the data and to form a perception of the whole. After that, the researcher identifies condensed meaning units, which are then transformed into main themes and grouped into categories of meanings.

## Results

The demographic information of the participants in the interviews is outlined in the table below (Table 1).

**Table 1:** Location and number of interviewees at each organization

Organization	Organizational Role	No. of Participants
Ministry of Health	Head of the Hospital Management Center	1
	Head of Development and Organizational Transformation	1
	Head of Insurance and Medical Economics Department	1
	Head of Health Information Technology	1
	Scientific and technological advisors of Research and Technology Vice-Chancellor	4
Health Insurance	Head of the Health Insurance Evaluation and Monitoring Group	1
	Strategic purchasing managers	2
	Treatment managers	2
	Head of Electronic Health Department	1
	Head of the Department of Chronic Diseases and Geriatrics	1
	Health insurance experts	1
Medical Informatics Association	Head of the Medical Informatics Association of the country	1
Medical Council	Directors of the Medical Council	2

In Continuing, after conducting 19 interviews, data saturation was achieved. Summary of the main and sub-themes of challenges and solutions is presented in the table below (Table 2).

**Table 2:** Summary of the main and sub-themes of challenges and solutions for implementing telemedicine in Iran

Category	Themes	Sub-Themes
**challenges**	Policy weakness in telemedicine	Attitude towards the provision of health services in the electronic platform
		Not being a priority in national programs
		The short life of management in the health system
	Uncertainty of telemedicine operating mechanisms	Unclearness of program management
		Lack of roadmap
		Lack of operational standards
		Inappropriate use of experts
	Lack of communication and telecommunication infrastructure	Unsuitability of internet infrastructure in the country
		Unsuitability of software infrastructure in the field of telemedicine
		Limited access to hardware in the field of telemedicine
	Lack of cultural infrastructure in telemedicine reception	Resistance in changing the service delivery model by service providers
		Resistance in accepting new service delivery models by society
		Insufficient literacy and skill in working with electronic systems
	Lack of electronic requirements	Inconsistency of patients’ clinical information and
		Weakness in transferring clinical data in the electronic platform
		Weakness in electronic health data management
	Redundant bureaucracies	Hard and complex laws
		Ambiguous and lengthy licensing process
	Legal gaps	Incompatibility of existing laws with the electronic world
		Ambiguity of professional and legal responsibilities
		lack of legal mechanism for authentication of service providers
		lack of legal mechanism for authentication of service recipients
	Economic factors	The high cost of setting up telemedicine
		the vagueness of the telemedicine service tariff and reimbursement mechanism
**Solutions**	National determination	Strengthening trust and belief at the governance level
		Predicting specific financial resources
		Coherent organization
		Involving experts and stakeholders
	Telemedicine roadmap setting	Development of economic studies
		Development of the program
		Composition of standards and regulations
		Gradual implementation of the program
		Implementation of the program in the context of family doctor and referral system
	Development of e-health requirements and infrastructure	Legislation
		Use of electronic health records
		Increasing the speed and accessibility of Internet
		Investment in the field of information technology
		Investment in the field of updating medical equipment
		Using the capacity of the private sector
	Preparing the community to accept telemedicine	Culturization and education in the field of telemedicine
		Introduction of telemedicine sciences in university education

The findings showed that telemedicine implementation in Iran faces eight main challenges, as follows:

### Policy weakness in telemedicine

This category encompasses “the type of attitude towards the provision of health services on the electronic platform,” “not being a priority in national programs,” and “the short life of management in the health system.”

The findings reveal that Iranian health authorities have not accorded telemedicine the necessary priority thus far. A participant articulated, “*Because they still don’t have a plan, they haven’t felt the need for it, and the health system and insurances haven’t recognized the necessity of telemedicine yet… because there is no national commitment for telemedicine*” (P 1).

This challenge is influenced by two critical factors: the attitude towards providing health services using telemedicine and the brief management periods in the health system. As one participant observed, “*Telemedicine usually requires long-term planning due to the short life of managers in our country. Managers usually prefer not to invest in projects that will not yield immediate results or will not be evaluated during their tenure. Long-term planning often yields little result in our country*” (P 2).

Moreover, the implementation of telemedicine demands substantial investment and long-term planning, with its cost-effectiveness potentially becoming evident only after several years. Consequently, health managers have hesitated to adopt it, and policymaking in this area has been deficient.

### Uncertainty of telemedicine operating mechanisms

Four sub-themes have been identified for this challenge: “unclear program management,” “lack of a roadmap,” “inappropriate use of experts,” and “lack of operational standards.”

The absence of a specific authority for policymaking in this domain has led to the abandonment of scattered projects due to the lack of coordination between different organizations and the absence of a clear roadmap. Additionally, the lack of comprehensive standards has resulted in the non-acceptance of these services by healthcare institutions, with organizations being hesitant to implement them due to numerous uncertainties.

Participants expressed the view that the inefficient use of experts and the failure to leverage the knowledge and experience of both domestic and foreign experts in this field underlie many other challenges. One participant highlighted, “*Unfortunately, there is currently no oversight for this issue. The deputy director of the Ministry of Health has entered with his own personal interests and desires, but he does not have a government order to mandate everyone to use telemedicine and carry it forward according to specific leadership. The Ministry of Health’s Information Technology department is not pursuing this business because it still has its own electronic version business*” (P3). Another participant emphasized, “*Unfortunately, the Ministry of Health is not living up to its responsibilities. As the policy maker of the country’s health system, the Ministry of Health should enter into these fields, define the standards, and establish conditions*” (P4).

Concerning the absence of a roadmap, a participant commented, “*There is no plan for the implementation of telemedicine in the country, meaning there is no such program in the country’s health system or insurance organizations… At least I don’t see one*…” (P5). Another participant highlighted the deficiency in necessary standards, stating, “*We don’t have regulations or standards in the field of telemedicine. We have overarching laws, but examples of how they apply to telemedicine are not clearly defined. There is no specific regulation for telemedicine*…” (P6).

### Lack of communication and telecommunication infrastructure

The participants identified several challenges in this domain, encompassing an inadequate internet infrastructure, insufficient software infrastructure for telemedicine, and restricted access to hardware.

The provision of high-speed and extensive internet is pivotal for the successful implementation of telemedicine; however, Iran confronts formidable challenges in this sphere. Communication lacks robustness, manifesting numerous impediments in data transfer to electronic systems. As articulated by one participant, “*To establish effective telemedicine, a robust communication infrastructure and internet are imperative. For instance, Iran is yet to adopt 5G internet technology. The current internet quality is suboptimal, impacting online consultations, which predominantly rely on text, and, at best, audio. Although attempts have been made to conduct video-based face-to-face consultations in various locations, the majority fall short of desired quality standards*” (P7).

Specialized telemedicine encounters several constraints attributable to the legal impossibility of utilizing external software and servers due to concerns regarding information confidentiality. The prevailing national software exhibits a rudimentary design with limited functionalities that inadequately align with the requisites of telemedicine across all levels. A participant highlighted this issue, stating, “*In our country, standards pertaining to patients’ prescriptions are still nonexistent. Consequently, when physicians attempt to employ software systems for prescribing medications, they encounter a formidable challenge; navigating through user-unfriendly software poses significant difficulties*” (P8).

Furthermore, an additional impediment to telemedicine lies in the scarcity of hardware and essential equipment required to deliver comprehensive services. This deficiency confines telemedicine activities to remote visits and consultations in Iran, impeding the seamless transfer of information. Another participant emphasized this challenge, elucidating, “*Patient care equipment equipped with data storage and transfer capabilities faces numerous challenges in Iran. The production, distribution, and accessibility of such equipment to various centers or individuals pose significant hurdles. In instances of illness, where continuous monitoring is essential, individuals should have access to equipment without the burden of personal acquisition costs. Establishing a system where individuals can obtain and utilize such equipment at home, returning it after use, becomes crucial. Failing to achieve this on a large scale poses a substantial obstacle to the widespread adoption of telemedicine*” (P18).

### Lack of cultural infrastructure in telemedicine reception

The challenges within this category encompass “Resistance in changing the service delivery model by service providers,” “Resistance in accepting new service delivery models by society,” and “insufficient literacy and skill in working with electronic systems.”

Physicians and healthcare providers exhibit resistance to transitioning from traditional to electronic service patterns, underscoring the unpreparedness of the cultural context necessary for the successful implementation of telemedicine. Policymakers highlight concerns such as the fear of misdiagnosis and treatment due to limited access to clinical information, the impossibility of physical examination, challenges in monitoring patients, and the potential mismatch of treatment plans prescribed by competent authorities with existing guidelines. Furthermore, the anticipated loss of jobs, including secretarial positions, is a notable apprehension. A policymaker emphasized this perspective, stating, “*Many care providers admitted that because it is not possible to directly visit the patient, touch the patient, ask the patient, examine the body parts, etc., therefore, telemedicine may not have the appropriate quality*” (P10). Another policymaker highlighted job-related resistance, noting, “*Many may disagree (especially in government systems) because the personnel, secretaries, etc. will disappear and the doctor will no longer need them, but because they will lose their jobs, one of the resistance factors for telemedicine*” (P11).

Also, the cultural context is not mature in the client community. The existence of traditional and religious beliefs, diversity of cultures in different parts of the country, and concerns about privacy violations are some of the issues introduced as challenges “*Depending on the type of service you receive. The basic challenge is the culture of the people and the culture of our medicine. That is, a doctor on this side must have seen telemedicine training, and a patient on the other side must be willing and ready to receive online consultations. have the line and distance*” (P12), “*We have ethnic, linguistic, cultural and religious diversity in the country, which have the ability to have multiple effects in the implementation of telemedicine, and we should pay attention to them*” (P13). In addition to the above, patients’ and physicians’ low literacy and ability to work with electronic systems (in all dimensions and not only technical literacy) are among the challenges showing that there has been no planned and coherent training in this area.

### Lack of electronic requirements

Within this category, challenges include the “Inconsistency of patients’ clinical information,” “Weakness in transferring clinical data in the electronic platform,” and “Weakness in electronic health data management.”

The inconsistency of patients’ clinical information, coupled with its non-integration into electronic health records, poses a limitation for healthcare providers in delivering diagnostic and treatment services. This inconsistency increases the likelihood of errors. A participant highlighted this issue, stating, “*Right now, even though there are 1034 hospitals in the country, they all have some kind of hospital information system software program in them, but right now we cannot centrally claim that all clinical data in the Ministry of Health. We have the patients at one place…”* (P10). Additionally, the continued reliance on paper for recording medical equipment usage in clinics and hospitals presents a challenge in transferring patient clinical data to telemedicine systems.

The lack of policy unity in e-health initiatives contributes to challenges in the transfer of clinical data between various organizations. Different organizations, each guided by the economic conditions and approaches of their health managers, have launched electronic systems projects independently, resulting in disparate systems. Consequently, the transfer of clinical data encounters numerous problems arising from this lack of standardization.

Another challenge in this realm is the shortage and high cost of IT specialists and big data management. The implementation of telemedicine is anticipated to generate a substantial volume of clinical data, necessitating proficiency in managing big data. The scarcity of skilled professionals and the associated high costs pose challenges in preparing and utilizing this data effectively for health policy purposes.

### Redundant bureaucracies

The category encompasses “rigorous and intricate laws” as well as an “ambiguous and protracted licensing process.” While stringent rules are essential to uphold the confidentiality of public health information, the combination of strict regulations and the requirement to obtain numerous unnecessary licenses at each telemedicine stage tends to practically deter healthcare providers from launching this service.

On the other hand, the licensing authority for the various stages of setting up telemedicine is not yet known, and multiple organizations claim to license in this area. So that specifying the steps and authorities is necessary *“Unfortunately, both our people and our health service providers do not have a good level of information technology literacy, and they do not feel motivated to increase this literacy”* (P 17).

### Legal gaps

The “incompatibility of existing laws with the electronic world,” the “ambiguity of professional and legal responsibilities,” the “lack of a legal mechanism for the authentication of service providers,” and the “absence of a legal mechanism for the authentication of service recipients” are encompassed within this category.

Various legal dilemmas have affected telemedicine implementation; failure to improve the rules in line with electronic advances is one. Execution of electronic prescription, electronic order, and electronic referral requires an electronic signature platform in national infrastructure and its recognition by law *“A large part of the laws that exist in the field of healthcare (such as doctor’s documents or patient information, which are part of the patient’s property and we are obliged to preserve them), are still in the paper world and have not yet been electronic and online, which causes many problems. It creates a trap for telemedicine”* (P 11). The use of two-factor passwords, due to the high probability of hacking, alone cannot provide security for service deliverers.

The next challenge is not determining professional responsibilities in health services deliveries, incredibly remote consulting services, whose duties are not legally specified. Therefore, In the event of a problem or a medical mistake, it is unclear whose fault it was *“There are ethical and legal issues, for example, if the patient’s information is leaked, who is to blame, if something happens to the patient, who is to blame? The doctor who gives advice remotely or the patient who receives advice has failed, these issues Because it has not been solved yet, the telemedicine system has remained at the same stage”* (P 3).

The lack of a legal mechanism for identifying employees is another issue that participants believed that if we could not define a precise legal mechanism, people would lose confidence in the system if even a few mistakes occurred, and the telemedicine acceptance would be delayed for a long time *“The method of authentication of doctors is important and it can be one of the obstacles that if one or two errors occur and people lose their trust because it is not known who is answering me, it may cause the whole work to stop and its development will be delayed”* (P16).

Policymakers have identified another critical legal gap: the absence of a legal mechanism for authenticating employees. Additionally, challenges persist in addressing the recording of unrealistic clinical information about patients in electronic health records and the potential misuse of insurance coverage, both of which lack comprehensive legal resolutions.

### Economic factors

“The high cost of setting up telemedicine” and “the vagueness of the telemedicine service tariff and reimbursement mechanism” are in this category.

Economic factors are one of the challenges that have always been worldwide, and more severe for developing countries like Iran. Telemedicine has now moved beyond virtual visiting and consulting to home care, so the high cost of initial telemedicine set-up and the need to invest in telemedicine and internet of things medical equipment is the first challenge to be addressed in this regard *“One part of it is the financial challenge. Considering the increase in currency prices and the sanctions we have, investing in information technology equipment is very expensive, and every organization that wants to go in this direction must make a very heavy investment in this. to have a domain”* (P1). The second challenge is the ambiguity of the tariff mechanism and reimbursement of telemedicine services due to the unclear standards and, insurance companies are concerned about increasing their costs by increasing the telemedicine deliveries. Also, the format of the contract with the insurance companies and the services that they cover are not clear to service providers *“If we cannot solve the issue of telemedicine tariff and its coverage by insurance, this service cannot develop much. It is possible that a group of people can use it who have the financial means and the tariff is too high for them. It doesn’t matter, but it will be difficult for the common people. There is not much supervision on the tariff, but the common people, especially those who are in the most deprived areas, and by the way, telemedicine can help them more, or they are in conditions such as the elderly., chronic patients and those whom telemedicine can help more, if this issue is not resolved, they will not be able to use and benefit from this service” (P5).*

### Solutions

Health policymakers in Iran have put four strategies aimed at accelerating and facilitating the implementation of telemedicine. These strategies include:

### National determination

“Strengthening trust and belief at the governance level”, “coherent organization”, involving experts and stakeholders” and “predicting specific financial resources” are in this category.

The first step in dealing with the challenges is to accept that telemedicine can be helpful in certain circumstances and to change the policy approaches of managers and officials as well. In the next step, all policy-making organizations in this field need to be under a coherent organization, and a single trustee should be considered for it, that can align the macro-policies of the organizations with implementing it by making trans-sectoral cooperation. On the other hand, involvement of experts and stakeholders helps in minimizing the planning errors and waste of resources. In the next step, it is necessary to consider specific financial resources to implement the program, providing by both governmental and private sectors *“The first thing is to believe that telemedicine exists at all, everyone in their own position in the health system, the Ministry of Health, the medical system, the insurance organizations, the program and budget organization and all the influencers of the country’s health system in the position They should come to believe that telemedicine exists and can be effective and useful for us, this is the most important factor for this to happen; then everyone should be able to use this capacity based on their own position. and identify and solve its challenges”* (P19).

### Telemedicine roadmap setting

“Development of economic studies”, “development of the program”, “composition of standards and regulations”, “gradual implementation of the program” and “implementation of the program in the context of family doctor and referral system” are included in this category. Findings showed that one of the effective ways to overcome the challenges is to set a roadmap. *“My suggestion is that if they want to go to telemedicine in our country, there should be a comprehensive program that will lead all health service providers to be electronic”* (P7). In the first step, it is necessary to conduct comprehensive economic studies in feasibility, needs assessment, and prioritization of its implementation. In the next step, a comprehensive plan that includes service standards and clear operational regulations is essential. Once the bars are determined, tariffs for services will help the health service providers practically. Implementing the program in the context of the referral system and family physician could significantly facilitate as well. The use of a referral system, especially in the follow-up of chronic diseases also deal many legal challenges due to the physician’s full knowledge of their covered population and the patient’s trust in the family physician.

### Development of e-health requirements and infrastructure

“Legislation”, “Use of electronic health records”, increasing the speed and accessibility of Internet lines”, “Investment in the field of information technology” and “Investment in the field of updating medical equipment” and “Using the capacity of the private sector” in They are placed in this category.

The implementation of telemedicine in Iran requires the simultaneous development of many infrastructures *“Before a doctor or treatment group wants to do something in the field of telemedicine, it is obvious that he must have access to the information and medical records, or in other words, the electronic health record of that patient; well, this does not exist in Iran;…; If the electronic infrastructure is solved, I think many problems will be solved”* (P12). First of all, legislation in various legal, ethical, and confidential dimensions is needed, that service providers are reluctant to use it if it is not resolved. The second is electronic health records, which can accelerate telemedicine implementation. It can be implemented in parallel with telemedicine implementation, too. Strengthening the national Internet infrastructure, investing in information technology and hardware and software development of this service needs attention. The next issue is investing in upgrades to generate digital costs and provide data transfer between medical equipment and electronic systems. Policymakers cited using the capacity of the private sector as an effective way to overcome government challenges in this matter.

### Preparing the community to accept telemedicine

“Culturization and education in the field of telemedicine” and “Introduction of telemedicine sciences in university education” are in this category.

The acceptance of telemedicine requires cultural maturity of coordination at all levels, both in the community of service providers and health care providers, which requires increasing the quality and quantity of training in this field. The findings also showed that creating an intellectual background and strengthening the knowledge and skills of telemedicine by introducing it in academic sciences can help prepare employees *“Like any new project, the government should create an opportunity for those interested and experts in this field to discuss and analyze the issue in the media and places that have a large audience…” (P18) and “Preparing the context for the acceptance of a new program depends on information…” (P17).*

## Discussion

The present condition of telemedicine in developing countries is deemed unsatisfactory and is undergoing a critical phase [2]. Developing nations have exhibited a sluggishness in embracing technology to mitigate healthcare expenses and enhance quality. Telemedicine, however, presents a promising avenue for surmounting challenges and augmenting access to health services [13].

Participants in this study expressed the viewpoint that implementing telemedicine constitutes a time-consuming mega-project, and the prevalence of short-term managerial tenures, coupled with a tendency among managers to favor expedient programs, represents a significant impediment. These findings are in concordance with the research conducted by Rafati and Molavi-Taleghani [14].

The absence of clear policymakers in the realm of telemedicine in Iran has resulted in a lack of specific standards within this domain. In contrast, countries such as India, with well-defined policies and facilitative measures, have successfully navigated the landscape of telemedicine development [15]. The dearth of defined criteria stands out as a fundamental challenge in the implementation of telemedicine, affecting physicians as service providers, insurance organizations, and individuals as service recipients. Additional research has pinpointed the absence of specific strategies, technical standards [14], and deficiencies in delineating the characteristics of components and operational processes [16] as notable challenges.

The telecommunication infrastructure in Iran, characterized by poor communication and telecommunication facilities, has impeded the seamless operation of telemedicine. Participants emphasized the critical reliance of telemedicine on high-speed internet and nationwide coverage for data exchange to occur in a timely and effective manner. Owing to weak internet connections, most telemedicine projects in the country rely on text and video, leading to interruptions in audio transmission. Furthermore, there is a notable absence of dedicated telemedicine hardware equipment and integrated software for streamlined data transfer. Studies underscore that insufficient information and communication technology infrastructure, encompassing hardware, software, and low internet bandwidth, act as impediments to telemedicine development in developing countries [17]. Mafi Moradi has similarly identified challenges in communication and information-related infrastructures, including non-qualified bandwidth, low internet speed, unclear audio and video in communications, the absence of portable platforms (such as tablets, personal digital assistants, and smartphones), and limited access to computers and related software [16]. The research conducted by Okoroafor et al. further substantiates the challenges posed by inadequate facilities and poor communication in the implementation of telemedicine in Africa [18].

The current study sheds light on the unpreparedness of Iran’s cultural infrastructure for the integration of telemedicine, a challenge shared by many other nations. Employee resistance to change, a pervasive issue discussed by Sagaro et al. in the context of Ethiopia [19], emerges as a critical factor influencing the sustainable implementation of telemedicine. Alboraie et al. [20] assert that physicians resist altering the established patterns of service delivery due to concerns about potential increases in medical errors, a sentiment echoed by Kruse et al. in their examination of telemedicine implementation challenges in Texas [21]. Similarly, Safdari et al.‘s study in Iran underscores the hindrance posed by the inability of some users to effectively utilize applications and equipment to the successful implementation of telemedicine [22].

The transformation of service delivery methods by telemedicine, coupled with cultural constraints among healthcare service providers, contributes to their resistance to adopting this technology [23]. Sagaro et al. [19] further identify a lack of user awareness as a barrier to telemedicine implementation in Ethiopia, while Kruse et al.‘s research [24] demonstrates that poor technical literacy among users presents a challenge to the implementation of telemedicine in the United States.

AlKureishi et al. highlight that patients’ lack of access to technology, skills, and unwillingness constitute barriers to the adoption of telemedicine [25]. Similarly, Ashfak et al.‘s study identifies poverty and lack of education as significant obstacles to telemedicine, particularly from the perspective of physicians in Karachi [26]. Consistent with these findings, the current study in Iran underscores the challenges posed by the lack of access to technology, skills, and economic constraints as reasons for the underutilization of telemedicine.

In the context of information management within telemedicine platforms in Iran, barriers include the absence of electronic health records, non-digital medical reports, and a shortage of IT specialists and big data infrastructure. The electronic health record is identified as a crucial component of the requisite information infrastructure for the successful establishment of telemedicine [27].

Lengthy bureaucratic processes and uncertainty regarding the formal issuing organization for telemedicine also emerge as challenges, a concern shared with Kruse et al.‘s observations in the United States [21]. Notably, the absence of a formal and specific mechanism and support to recognize electronic services, particularly within Iran’s healthcare system, exacerbates these challenges. Identified weaknesses encompass the lack of clear legal protocols in times of potential issues, the absence of authentication centers and digital signatures, and challenges in defining physicians’ responsibilities for their performance [16]. The collaborative aspect of telemedicine faces hurdles in Iran, with insufficient cooperation among involved organizations and ambiguity in their missions identified as significant obstacles [28].

Economic challenges and the high costs associated with establishing telemedicine services are pervasive not only in Iran but also in other countries. Given the diverse priorities within the health systems of developing nations, financing telemedicine activities is likely to remain a formidable challenge in the future [29]. However, avenues such as funding from foreign aid agencies or the private sector hold promise in addressing this challenge.

Sagaro et al.‘s study in Ethiopia underscores cost-related factors as the most significant obstacles to telemedicine implementation [19]. Similarly, Kruse et al. note the escalating costs of healthcare and limitations on repayments as challenges in the implementation of telemedicine in the United States [24]. Alaboudi et al. emphasize the importance of sufficient and sustainable financial support for the implementation, operation, maintenance, and reimbursement of telemedicine systems [30]. Kruse et al.‘s findings in Texas also highlight costs and reimbursement as barriers to telemedicine [21].

Despite these challenges, it is crucial to recognize that telemedicine has the potential to substantially reduce overall healthcare costs by fundamentally restructuring the delivery of healthcare services. This transformative potential makes addressing economic challenges a critical aspect of realizing the broader benefits of telemedicine.

### Recommendations

Initiating telemedicine in developing countries requires a multifaceted approach, beginning with the dissemination of information and education to policymakers regarding its potential benefits in addressing medical and healthcare shortages. Conducting small-scale pilot projects is recommended to draw attention to these benefits and familiarize policymakers with the challenges inherent in telemedicine implementation. Additionally, studies focusing on the cost-effectiveness of telemedicine in developing countries, such as Iran, are deemed necessary. Overcoming existing challenges can be facilitated by strengthening policymakers’ belief in the benefits of telemedicine and providing adequate financial support, strategies which have been identified as effective [31, 32].

The establishment of a national digital health roadmap emerges as a crucial strategy for overcoming challenges associated with telemedicine implementation. This approach has proven effectiveness in addressing various hurdles. Previous research emphasizes the significance of organizational factors and strategic planning in the successful implementation of telemedicine [33]. Additionally, formulating a corporate strategy that benefits stakeholders has been identified as a key component [34].

In developing a national digital health roadmap, it is essential to set clear goals, outline plans to achieve them, and establish standards and guidelines in all dimensions. This comprehensive planning framework provides a structured and organized approach to navigate the complexities of telemedicine implementation. Moreover, the development of e-health requirements and infrastructure across communication, technical, and legal aspects should be integral to the roadmap. Past studies have consistently suggested the development of communication infrastructure and information technology as pivotal solutions in this regard [22, 31–33].

The current study underscores the significance of cultural and educational interventions as essential components in promoting the acceptance of telemedicine within the community. This aligns with findings from other research studies. Kruse et al. identified technology barriers and recommended change management-related education as a means to overcome these obstacles [21]. Similarly, Rezai’s study emphasized the importance of educating specialist physicians to eliminate barriers and facilitate the implementation of telemedicine [35]. AlKureishi et al. also highlighted physician education and the necessity of dismantling barriers between physicians and patients as viable solutions [25]. The consistency of these findings with the present study reinforces the critical role of education and cultural interventions in fostering acceptance and successful implementation of telemedicine initiatives.

In summary, a holistic approach that includes greater education and information dissemination to policymakers, the establishment of a national digital health roadmap, and investments in communication infrastructure and information technology holds significant potential in addressing the challenges associated with telemedicine implementation in developing countries. Concurrently, cultural and educational interventions are vital for garnering community acceptance and ensuring the successful integration of telemedicine into healthcare systems.

## Conclusions

From the perspective of health policymakers, the implementation of telemedicine in Iran presents significant and multidimensional challenges. These challenges span issues within the national healthcare system as well as those arising from policy-related institutions and organizations, creating a complex landscape for the successful execution of telemedicine initiatives. Overcoming these difficulties necessitates extensive inter-organizational cooperation and program leadership at the governance level. This leadership is crucial for directing policies across different organizations and effectively addressing the diverse challenges associated with telemedicine implementation.

It is important to recognize that conditioning the implementation of telemedicine on the complete elimination of challenges can be a time-consuming process and may potentially deprive the national health system and society of the benefits offered by telemedicine. Therefore, a more pragmatic approach involves leveraging recommendations derived from scientific and practical experiences within various positions in the healthcare field. By doing so, policymakers can accelerate the implementation of telemedicine, utilizing insights gained from real-world applications and scholarly knowledge.

In summary, the successful execution of telemedicine in Iran requires collaborative efforts and leadership at multiple levels. Policymakers must have a deep understanding of the challenges involved and leverage recommendations rooted in scientific and practical experiences to navigate and address these challenges effectively. This approach ensures a more expedited and pragmatic implementation of telemedicine, ultimately benefiting the national health system and society.

### Electronic supplementary material

Below is the link to the electronic supplementary material.

Supplementary Material 1

Supplementary Material 2

## Data Availability

As the responsible author of the present study, Mrs. Shiva Abdolahnejad Boushehri possesses the data and information related to it. Those who are interested in accessing this information may contact her directly.

## References

[1] Khemapech I, Sansrimahachai W, Toachoodee M (2019). Telemedicine–meaning, challenges and opportunities. Siriraj Med J.

[2] Bali S. Enhancing the reach of health care through telemedicine: Status and new possibilities in developing countries. Health care delivery and clinical science: concepts, methodologies, tools, and applications. IGI Global; 2018;1382–97.

[3] Wade V, Eliott J (2012). The role of the champion in telehealth service development: a qualitative analysis. J Telemed Telecare.

[4] Lowe AA, Gerald JK, Clemens C, Brown MA, Moore M, Carr TF, Care and Reducing Disparities in Asthma and Sleep. Mobile telemedicine programs in school could increase clinician efficiency and improve access to health care among children with Asthma. D14 IMPROVING. American Thoracic Society; 2016;A6427–A.

[5] Merchant K, Ward MM, Mueller KJ (2015). Hospital views of factors affecting telemedicine use. Rural Policy Brief.

[6] Kane CK, Gillis K (2018). The use of telemedicine by physicians: still the exception rather than the rule. Health Aff.

[7] FH Healthcare Indicators and the FH Medical Price Index. : An Annual view of place of service Trends and medical Pricing2019. Available from: https://www.s3.amazonaws.com/media2.fairhealth.org/whitepaper/asset/FH%20Healthcare%20Indicators %20and%20FH%20Medical%20Price%20Index%202019%20-%20A% 20FAIR%20Health%20White%20Paper.pdf. Accessed November 1, 2020.

[8] Standing C, Standing S, McDermott ML, Gururajan R, Kiani Mavi R (2018). The paradoxes of telehealth: a review of the literature 2000–2015. Syst Res Behav Sci.

[9] Saffura E, mohhamad ZS. Identification and prioritization of barriers to the use of telemedicine technology in medical centers (case study: Shahid Beheshti University of Medical Sciences. The first congress of applied research in Industrial Managment of Azad University of Semnan; 2015.

[10] Organization WH. World health statistics 2020: monitoring helath for the SDGs, sustainable development goals. Geneva. 2020.

[11] Bashshur R, Doarn CR, Frenk JM, Kvedar JC, Woolliscroft JO. Telemedicine and the COVID-19 pandemic, lessons for the future. Mary Ann Liebert, Inc., publishers 140 Huguenot Street, 3rd Floor New … p. 571-3. 10.1089/tmj.2020.29040.rb 32275485

[12] Erlingsson C, Brysiewicz P (2017). A hands-on guide to doing content analysis. Afr J Emerg Med.

[13] Bali S. Barriers to development of telemedicine in developing countries. InTelehealth 2018 Dec 5. IntechOpen.

[14] Rafati H, Molavi Taleghani Y (2019). Feasibility study for the establishment of Telemedicine: a review study and a suggestion for Iran. J Health Biomedical Inf.

[15] Pal A, Mbarika VW, Cobb-Payton F, Datta P, McCoy S (2005). Telemedicine diffusion in a developing country: the case of India (March 2004). IEEE Trans Inf Technol Biomed.

[16] Mafi-Moradi S, Doshmangir L, Kabiri N (2019). Challenges and opportunities of Telemedicine: a narrative review study. Health Inf Manage.

[17] Hassibian MR, Hassibian S. Telemedicine acceptance and implementation in developing countries: benefits, categories, and barriers. Razavi Int J Med. 2016;4(3).

[18] Okoroafor I, Chukwuneke F, Ifebunandu N, Onyeka T, Ekwueme C, Agwuna K (2017). Telemedicine and biomedical care in Africa: prospects and challenges. Niger J Clin Pract.

[19] Sagaro GG, Battineni G, Amenta F (2020). Barriers to sustainable telemedicine implementation in Ethiopia: a systematic review. Telemedicine Rep.

[20] Alboraie M, Allam MA, Youssef N, Abdalgaber M, El-Raey F, Abdeen N et al. Knowledge, Applicability, and Barriers of Telemedicine in Egypt: A National Survey. International Journal of Telemedicine and Applications. 2021. 10.1155/2021/5565652 PMC8192215 34211550

[21] Kruse C, Karem P, Shifflett K, Vegi L, Ravi K, Brooks M. Evaluating barriers to adopting Telemedicine worldwide. A systematic review. 10.1177/1357633X16674087 PMC5768250 29320966

[22] Safdari R, Gholamzadeh M, Rezayi S, Tanhapour M, Saeedi S. Telehealth and Telemedicine in response to critical coronavirus: a systematic review. Iran Red Crescent Med J. 2021;23(9).

[23] Saghaeiannejad-Isfahany S, Jahanbakhsh M, Shayan A (2015). Telemedicine operational feasibility in selected hospitals of Isfahan University of Medical Sciences in 1393. Int J Health Syst Disaster Manage.

[24] Kruse CS, Williams K, Bohls J, Shamsi W (2021). Telemedicine and health policy: a systematic review. Health Policy and Technology.

[25] Alkureishi MA, Choo Z-Y, Lenti G, Castaneda J, Zhu M, Nunes K (2021). Clinician perspectives on telemedicine: observational cross-sectional study. JMIR Hum Factors.

[26] Ashfaq A, Memon SF, Zehra A, Barry S, Jawed H, Akhtar M et al. Knowledge and attitude regarding telemedicine among doctors in Karachi. Cureus. 2020;12(2). 10.7759/cureus.6927 PMC7065727 32190480

[27] Mirani N, Ayatollahi H, Haghani H. A survey on barriers to the development and adoption of electronic health records in Iran. Journal of Health Administration (JHA). 2013;15(50).

[28] Khalifehsoltani SN, Gerami MR. E-health challenges, opportunities and experiences of developing countries. In2010 International Conference on e-Education, e-Business, e-Management and e-Learning 2010 Jan 22 (pp. 264–268). IEEE.

[29] Wright D (1998). Telemedicine and developing countries. A report of study group 2 of the ITU Development Sector. J Telemed Telecare.

[30] Alaboudi A, Atkins A, Sharp B, Balkhair A, Alzahrani M, Sunbul T (2016). Barriers and challenges in adopting Saudi telemedicine network: the perceptions of decision makers of healthcare facilities in Saudi Arabia. J Infect Public Health.

[31] Ghaemi SA, Dong H, Alizada M, Hayatullah G. State of Telemedicine and Teledermatology in Iran: A Review Of Literature.

[32] Mashoof Ahmadimmerajim, Paramedical Science and Rehabilitation (JPSR). E. Evidence on Telemedicine in Iran-systematic review. Volume 7. JOURNAL OF; 2018. pp. 112–24. [cited 2022April05];. 1.

[33] Azizi A, Sharifat Z (2017). Barriers to Telemedicine: perspectives of the administrators. Front Health Inf.

[34] Khodadad-Saryazdi A (2021). Exploring the telemedicine implementation challenges through the process innovation approach: a case study research in the French healthcare sector. Technovation.

[35] Rezaei P, Maserrat E, Torab-Miandoab A (2018). Specialist Physicians’ perspectives about Telemedicine and barriers to using it in Tabriz Teaching hospitals. Iran South Med J.

