# Supplementary material for: Challenges and solutions for implementing telemedicine in Iran from health policymakers’ perspective
Source: BMC Health Serv Res. 2024 Jan 10;24:50. doi: 10.1186/s12913-023-10488-6 (PMC10782789; doi:10.1186/s12913-023-10488-6)
Supplement: Supplementary file 1 — Supplementary Material 1 [file 12913_2023_10488_MOESM1_ESM.docx]

Table 1 Consolidated criteria for reporting qualitative studies (COREQ): a 32-item checklist

**No Item Guide questions*/*description**

. ... ... ... ... ... .. ... ... ... ... ... ... ... ... ... ... ... ... ... ... ... ... .. ... ... ... ... ... ... ... ... ... ... ... ... ... ... ... ... .. ... ... ...... ... ... ... ... ... ... ... ... ... ... ... ... .. ... ..

**Domain 1: Research team and reflexivity**

***Personal Characteristics***

1. **Interviewer*/*facilitator** Shiva Abdolahnejad Boushehri
2. **Credentials** Graduated with a master's degree in health management
3. **Occupation** Quality improvement manager of Razi Hospital, Tehran University of Medical Sciences
4. **Gender** Female
5. **Experience and training Relationship with participants** Quality improvement manager of Razi Hospital, Tehran University of Medical Sciences and has some experiences doing interviews.
6. **Relationship established** S AB, SM H and K A were involved in the study design, data collection, data entry, analysis, and drafted the original manuscript. S AB was involved in translating the manuscript.
7. **Participant knowledge of the interviewer**

A brief explanation of the study consisting of the research question, objectives and the questions have been sent to participants after setting the interview data and time.

1. **Interviewer characteristics** All researchers have no conflict of interest in the study field; they were aware of the literature and interested in the issue.

**Domain 2: study design**

***Theoretical framework***

1. **Methodological orientation and Theory**

***Participant selection***

Content analysis

1. **Sampling** Both purposive and snowball
2. **Method of approach** face-to-face
3. **Sample size** 19
4. **Non-participation** two candidates were omitted because they were busy and postponed the interview more than two times.
5. ***Setting***
6. **Setting of data collection** Workplace
7. **Presence of non-participants** No
8. **Description of sample** A total of 19 policymakers participated from health service providers, including the treatment, hygiene, development, research, and technology deputies of the health ministry and its affiliated universities of medical sciences, Health Insurance Organization, and National Medical Informatics Association.

***Data collection***

1. Interview guide Researchers have set the potential steps of interviewing as a guide for both open and semi-structured forms of interviewing
2. Repeat interviews No
3. Audio*/*visual recording The interviews have been recorded using a call recorder, screen recorder, or voice recorder software and apps.
4. Field notes Yes. The records were transcript completely, but during the interview, the researcher used notes, especially for the in-depth questions.
5. Duration The duration of the interviews varied from 20 to 85 minutes.
6. Data saturation Yes
7. Transcripts returned No

Domain 3: analysis and ﬁndings

Data analysis

1. Number of data coders 1
2. Description of the coding tree: The authors described the coding individually and then discussed it in a session
3. Derivation of themes The themes are based of qualitative studies.
4. Software MAXQDA 2018
5. Participant checking
6. The initial form of the codes and themes has been checked with the third researcher
7. Quotations presented The main quotations has been mentioned for each code in the manuscript.
8. Data and findings consistent Yes
9. Clarity of major themes Yes
10. Clarity of minor themes Yes

(ii) Participant selection: Researchers should report how participants were selected. Usually, purposive sampling is used, which involves selecting participants who share particular characteristics and have the potential to provide rich, relevant, and diverse data pertinent to the research question

1. A total of 19 policymakers participated from health service providers, including the treatment, hygiene, development, research, and technology deputies of the health ministry and its affiliated universities of medical sciences, Health Insurance Organization, and National Medical Informatics Association.
