# Supplementary material for: Challenges and solutions for implementing telemedicine in Iran from health policymakers’ perspective
Source: BMC Health Serv Res. 2024 Jan 10;24:50. doi: 10.1186/s12913-023-10488-6 (PMC10782789; doi:10.1186/s12913-023-10488-6)
Supplement: Supplementary file 2 — Supplementary Material 2 [file 12913_2023_10488_MOESM2_ESM.docx]

**Interview Guide**

The interview questions include:

1. What is the current state of telemedicine implementation in the country?
2. What has been Iran’s experience with telemedicine implementation so far?
3. What obstacles do you currently consider to be hindering the implementation of telemedicine in the country?
4. What are the challenges related to organizational factors?
5. What are the challenges related to the infrastructure and technical requirements of this project?
6. What are the challenges in the field of legal issues?
7. What human-related problems are involved in implementing telemedicine in the country?
8. What requirements do you consider necessary to address the existing challenges?
9. Is there another matter that hasn’t been mentioned in these questions but needs to be addressed?

Thank you very much for your attention and cooperation.
